# Degradation Modeling and RUL Prediction of Hot Rolling Work Rolls Based on Improved Wiener Process

**DOI:** 10.3390/ma17204943

**Published:** 2024-10-10

**Authors:** Xuguo Yan, Shiyang Zhou, Huan Zhang, Cancan Yi

**Affiliations:** 1 Key Laboratory of Metallurgical Equipment and Control Technology, Ministry of Education, Wuhan University of Science and Technology, Wuhan 430081, China; yanxuguo@wust.edu.cn (X.Y.); zhanghuan@wust.edu.cn (H.Z.); meyicancan@wust.edu.cn (C.Y.); 2 Hubei Key Laboratory of Mechanical Transmission and Manufacturing Engineering, Wuhan University of Science and Technology, Wuhan 430081, China; 3 Precision Manufacturing Institute, Wuhan University of Science and Technology, Wuhan 430081, China

**Keywords:** remaining useful life prediction, degradation modeling, Wiener process, pulsed eddy current testing, hot rolling work rolls

## Abstract

Hot rolling work rolls are essential components in the hot rolling process. However, they are subjected to high temperatures, alternating stress, and wear under prolonged and complex working conditions. Due to these factors, the surface of the work rolls gradually degrades, which significantly impacts the quality of the final product. This paper presents an improved degradation model based on the Wiener process for predicting the remaining useful life (RUL) of hot rolling work rolls, addressing the critical need for accurate and reliable RUL estimation to optimize maintenance strategies and ensure operational efficiency in industrial settings. The proposed model integrates pulsed eddy current testing with VMD-Hilbert feature extraction and incorporates a Gaussian kernel into the standard Wiener process to effectively capture complex degradation paths. A Bayesian framework is employed for parameter estimation, enhancing the model’s adaptability in real-time prediction scenarios. The experimental results validate the superiority of the proposed method, demonstrating reductions in RMSE by approximately 85.47% and 41.20% compared to the exponential Wiener process and the RVM model based on a Gaussian kernel, respectively, along with improvements in the coefficient of determination (CD) by 121% and 19.76%. Additionally, the model achieves reductions in MAE by 85.66% and 42.61%, confirming its enhanced predictive accuracy and robustness. Compared to other algorithms from the related literature, the proposed model consistently delivers higher prediction accuracy, with most RUL predictions falling within the 20% confidence interval. These findings highlight the model’s potential as a reliable tool for real-time RUL prediction in industrial applications.

## 1. Introduction

Hot rolling work rolls serve as essential components and large consumable tools in the hot rolling process. Nevertheless, under long and complex working conditions, they are exposed to high temperatures, alternating stress, and wear [1]. As a consequence, the surface of work rolls undergoes gradual degradation, which has a serious impact on the quality of the final product [2]. Constructing a degradation model and realizing accurate prediction of the remaining useful life (RUL) of work rolls are of crucial significance to enhancing the service life of rolls and product quality [3]. This enables firms to rationally arrange the replacement and grinding times of work rolls in accordance with prediction results. Moreover, it helps avoid waste caused by premature replacement and prevents production interruptions, product quality issues, and additional maintenance costs due to delayed replacement. However, the degradation process of work rolls is influenced by multiple elements including the material of rolled products, the rolling process, the rolling force distribution, and the external environment. The degradation is intricate and hard to predict, exhibiting characteristics such as randomness, non-linearity, and strong coupling [4,5]. Consequently, constructing an accurate roll degradation model presents a significant challenge in this particular context.

In general, the existing research on roll degradation modeling mainly includes two types of methods: physical analytical models and data-driven methods [6]. The physical analytical models mainly construct mathematical models of roll degradation and predict the RUL by investigating factors influencing degradation such as roll material properties, failure mechanisms, and loading conditions. Cai et al. [7] indicated that the roll degradation process is affected by various elements including rolling force, strip width, temperature, rolling speed, and cooling water. These elements are interrelated and accompanied by mechanical wear, thermal wear, chemical corrosion, and other forms of degradation. On this basis, some research has further proposed improved models by considering elements such as roll surface temperature [8], rolling speed [9], and non-uniform wear [10]. Thus, it can be observed that roll wearing is a complex process involving multiple disciplines such as physics, chemistry, and material science. Simultaneously, the production process and operating state of work rolls are increasingly diverse, which makes the degradation modeling strategy based on physical analytical models still face challenges in meeting the new development needs of engineering practice.

Recently, data-driven methods have gradually become the mainstream direction for RUL prediction. To ensure the accuracy of prediction, obtaining a substantial amount of degradation data is crucial. The non-destructive testing technology provides an effective means for real-time acquisition of roll surface degradation data [11,12]. Three commonly used non-destructive testing methods include electromagnetic testing, ultrasonic testing, and radiographic testing. Pulsed eddy current testing is a new electromagnetic testing method that offers high detection efficiency, a wide range of frequency components, and good penetration. It can meet the requirement for online roll operating status monitoring [13,14,15]. In non-destructive testing, signal analysis plays a vital role in extracting information related to the degradation state of components or equipment. Common signal analysis methods include time-domain, frequency-domain, and time-frequency domain analyses [16]. Time-domain methods primarily focus on changes in signal amplitude and duration, providing insights into the overall signal characteristics. Frequency-domain methods, such as Fourier analysis, allow for the identification of dominant frequency components, which is useful for understanding specific degradation behaviors. Time-frequency domain analysis, such as Hilbert–Huang Transform (HHT), combines the advantages of both time and frequency domains, enabling the analysis of non-linear and non-stationary signals [17]. These methods allow for the extraction of features from signals, which can characterize the condition of materials or components, including defect detection, material damage, and corrosion levels. The extracted features can then be integrated with machine learning algorithms (such as neural networks, support vector machines, etc.) or degradation models (such as the Wiener process, Gamma process, etc.) to achieve accurate RUL predictions. For instance, features obtained through pulsed eddy current testing, such as signal marginal spectrum energy, can serve as inputs for data-driven models to map the relationship between degradation states and RUL.

Recently, only a few studies have utilized neural networks to simulate the degradation process and predict the RUL. Wu et al. [18] proposed a backpropagation neural network and finite element analysis software to obtain high-precision rolling force and accurate RUL prediction results by predicting the friction factors and others. Jiao et al. [19] introduced a new deep learning network based on recurrent neural networks to extract the health indicators from batch data to predict the RUL of rolls. However, the neural network requires a large amount of historical data to train the model; otherwise, it is difficult to predict the RUL accurately. Furthermore, the neural network models make it difficult to capture the randomness inherent in degradation data. Generally, they can only provide estimates of relevant characteristic quantities and are unable to represent the uncertainty of the degradation process.

In the process of roll degradation modeling, due to the influence of factors such as complex working conditions, the indicators employed to characterize the degradation of rolls will exhibit a certain degree of randomness as the working time increases. The stochastic process model (such as Wiener, Gamma, Inverse Gaussian, etc.) can effectively describe the uncertainty of degradation process over time and has been widely applied in engineering practice [20]. Among these models, the Wiener process is more appropriate for modeling non-monotonic degradation paths and has increasingly become one of the important methods in fundamental research and practical applications [21]. For instance, Chen et al. [22] proposed a degradation model based on the Wiener process. This model has the capability to effectively capture the degradation trends from various degradation data and subsequently accurately predict the RUL of batteries. Hu et al. [23] proposed an RUL prediction model based on the temperature parameters and successfully applied it for the RUL prediction of a wind turbine generator set. Compared with neural networks and other artificial intelligence methods, the Wiener process has the ability to handle uncertain information such as sample differences, measurement errors, environmental interference, etc. The failure distribution derived from the Wiener process incorporates the concept of first passage time. The concept can be utilized by firms to develop rational preventative maintenance plans and provides a quantitative analysis tool for realizing the degradation process and making proper decisions regarding maintenance strategies.

In this paper, with pulsed eddy current testing as the main technique, a degradation modeling and RUL prediction method of work rolls is proposed by introducing a kernel function into the standard Wiener process. Our method has the following contributions:

1. It combines pulsed eddy current testing technology with a real-time prediction model, enabling a comprehensive acquisition of degradation information and an effective characterization of the roll surface degradation state;
2. By introducing the Gaussian kernel function into the standard Wiener process, the improved model can handle uncertain information, effectively capturing the randomness and nonlinearity of the roll degradation process;
3. By constructing the probability density function (PDF) of the RUL, the method quantifies the uncertainty level associated with the roll degradation state, providing a valuable reference for RUL prediction and real-time equipment monitoring in other similar fields.

In summary, the novelty of this paper lies in the integration of the Gaussian kernel function into an improved Wiener process, combined with VMD-Hilbert feature extraction and a Bayesian framework, which, together, enable accurate modeling and RUL prediction of the nonlinear degradation paths of hot rolling work rolls. This approach provides a more robust and versatile solution for roll degradation analysis than existing methods, marking a significant advancement in the field.

The remainder of this paper is organized as follows. Section 2 gives an overview of the proposed method and the fundamental theories on which it is based. Section 3 explains the proposed improved method in detail, including the construction of roll degradation model based on the improved Wiener process, the estimation of unknown parameters based on the Bayesian framework, and the RUL prediction of work rolls. Section 4 outlines the experimental plan and preliminary preparation for the RUL prediction of work rolls in actual field conditions, including the extraction of feature parameters and the selection of the kernel function. Section 5 presents the experimental results of RUL prediction and discusses comparisons with other related algorithms to demonstrate the effectiveness and practicality of the proposed method. The conclusion and future work are drawn in Section 6.

## 2. Fundamental Theories

### 2.1. Pulsed Eddy Current Testing

**Figure 1:**
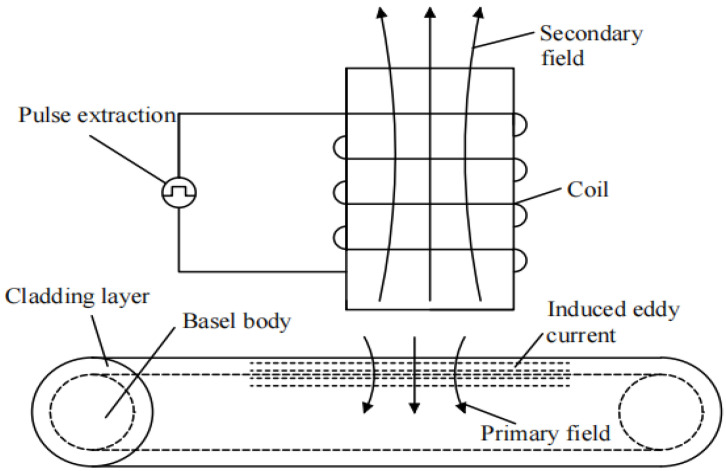
The principle of pulsed eddy current testing.

Pulsed eddy current testing is a non-destructive tool used to identify the defects and changes in the surface of metal materials. It is particularly valuable in assessing the roll degradation. Figure 1 depicts the principle of pulsed eddy current testing. It works on the principle of using a pulsed electromagnetic field. A coil is used to generate a short-duration pulse of alternating current, which creates a magnetic field (the primary field). When the magnetic field is applied to a test object, it induces eddy current within the object (the secondary field).

The presence of defects or variations in the material properties of the test object (e.g., the work rolls) will affect the eddy current. These changes in the eddy current can be detected by measuring the response of magnetic field or by analyzing the characteristics of induced currents. By analyzing the measured signals, it is possible to determine the location, size, and defect types in the tested roll. It can also be used to assess the degree of roll degradation, providing an important basis for roll maintenance and replacement.

### 2.2. Time-Frequency Analysis

The generation and variation of pulsed eddy current signal are related to multiple features of the test object. In the signal analysis process, various mathematical methods and tools are generally used to extract and interpret this information. Commonly used methods include time-domain analysis, frequency-domain analysis, and time-frequency domain analysis. Since pulsed eddy current signals are a typical non-stationary signal, in order to retain the time information in the signal, the time-frequency domain analysis is adopted in this paper. The Hilbert–Huang Transform (HHT) is a method for analyzing non-linear and non-stationary time series proposed by Nordene E. Huang et al. [24]. This method mainly consists of two parts: Empirical Mode Decomposition (EMD) and Hilbert Spectrum Analysis (HSA). However, in EMD, the model aliasing problem may occur, leading to unstable decomposition and the generation of new Intrinsic Mode Functions (IMF).

To solve the above problem, this paper combines Variational Mode Decomposition (VMD) with HHT and proposes a VMD-Hilbert method to decompose a complex signal into multiple IMF components with different center frequencies. The advantage of this combination lies in its ability to adaptively decompose a signal into modes of different frequencies and simultaneously obtain the instantaneous frequency information of each mode. It can analyze the time-frequency features of non-stationary signals with greater accuracy and exhibit better noise robustness as well as fewer model-aliasing problems. The general procedures of the VMD-Hilbert method are as follows:

1. The original signal is first decomposed using Variational Mode Decomposition (VMD) to obtain a series of Intrinsic Mode Function (IMF) components;
2. Hilbert–Huang Transform (HHT) is then performed on each IMF component to extract its instantaneous frequency and instantaneous amplitude;
3. By analyzing the changes in instantaneous frequency and instantaneous amplitude, the feature information of the signal in the time-frequency domain is obtained, revealing the transformation of different frequency components over time.

Let the number of IMF components obtained after decomposition be M, and its constrained variational model is as follows:

(1) $$\underset{\left\{\mu_{k}\right\},\left\{\omega_{k}\right\}}{\min}\left\{\sum_{k=1}^{M}{\left[{𝜕}_{t}\left(\left(\delta \left(t\right)+\frac{j}{\pi t}\right)*\mu_{k}\left(t\right)\right)e^{-j\omega_{k}t}\right]}_{2}^{2}\right\}\;s.t.\sum_{k=1}^{M}\mu_{k}\left(t\right)=f\left(t\right)$$

where δ is the Dirac distribution, $\mu_{k}$ is the IMF obtained after decomposition, $\omega_{k}$ is the center frequency of each IMF, $f\left(t\right)$ is the original pulsed eddy current signal, δ represents the gradient calculation, and ∗ represents the convolution calculation.

Perform HHT on the IMF to obtain the Hilbert spectrum of the pulsed eddy current signal. Integrate the Hilbert spectrum along the time axis to transform its relationship among amplitude, time, and frequency into a relationship between amplitude and frequency, thus describing the distribution of amplitude (or energy) on the frequency axis. Compared with Fourier spectrum, the Hilbert marginal spectrum has a certain degree of probabilistic significance, can be regarded as a weighted joint amplitude–frequency–time distribution. The weight assigned to each time–frequency unit is the local amplitude. In the Hilbert marginal spectrum, the existence of energy at a certain frequency implies the possibility of the existence of vibration with that frequency, and the specific moment when this vibration occurs is given in Hilbert spectrum. When processing non-stationary signals, the marginal spectrum is more applicable than the Fourier spectrum. This is because, in order to mathematically fit the non-stationary waveform of the original data, Fourier transform has to introduce a large number of high-frequency “pseudo” harmonic components, which will lead to an underestimation of low-frequency energy by the Fourier spectrum. Therefore, the marginal spectrum is more suitable for processing pulsed eddy current signals.

The expression of the Hilbert marginal spectrum is as follows:

(2) $$h\left(\omega \right)=\int_{0-\infty }^{T\infty }H\left(\omega ,t\right)dt$$

where $H\left(\omega ,t\right)$ is the Hilbert spectrum, which represents the amplitude of the signal at time t and frequency ω.

Multiple features can be extracted from the Hilbert marginal spectrum, specifically including the following:

1. Amplitude–Frequency Distribution: describes the distribution of signal amplitude (or energy) at different frequencies, helping to understand the degree of energy concentration and distribution rules at each frequency;
2. Marginal Spectrum Energy: reflects the energy magnitude of the signal at different frequencies and is used to compare the relative importance of different frequency components;
3. Centroid: represents the central position of spectral energy distribution, obtained by calculating the weighted average of frequency and corresponding amplitude, and can reflect the overall shift of signal frequency characteristics;
4. Bandwidth: describes the width of the frequency interval where the signal energy is mainly concentrated. A wider bandwidth may indicate that the signal contains a wider range of frequency components, while a narrower bandwidth means that the frequency components are relatively concentrated;
5. Amplitude at Specific Frequencies: focuses on the amplitude at certain specific frequencies, and these specific frequencies may be related to certain characteristics or faults of the analyzed object.
6. Changing Trend of Energy Distribution: observes how energy distribution changes as the frequency changes, such as whether there are obvious peaks, valleys, or slope changes, etc., which can provide information about the dynamic characteristics of the signal.

Compared with other features, marginal spectrum energy can provide an overall quantitative description of signal energy, intuitively compares total energy differences of different signals or the same signal in different states, and helps to quickly determine the main concentrated area of energy in the signal and grasp macroscopic characteristics of energy distribution. Therefore, marginal spectrum energy can be used to compare the energy differences between normal and faulty states and determine whether faults cause significant changes in energy. It is more suitable for extracting roll degradation features. The expression of marginal spectrum energy is as follows:

(3) $$M\left(\omega \right)=\int_{-\infty }^{\infty }H\left(\omega ,t\right)dt$$

where $M\left(\omega \right)$ represents the marginal spectrum, ω represents frequency, t represents time, and $H\left(\omega ,t\right)$ represents the Hilbert spectrum.

In discrete signals, the above integral formula can be changed to an approximate summation form:

(4) $$M\left(\omega \right)\approx \sum_{k}H\left(\omega ,k\right)\cdot ∆t$$

where $H\left(\omega ,k\right)$ is the time–frequency spectrum matrix and ∆t is the time interval.

Simultaneously, in order to minimize the differences among different individuals, the increment of marginal spectrum energy and weighted value of marginal spectrum energy can be calculated to more accurately depict the degradation process of rolls.

## 3. The Proposed Method

### 3.1. Methodology Overview

**Figure 2:**
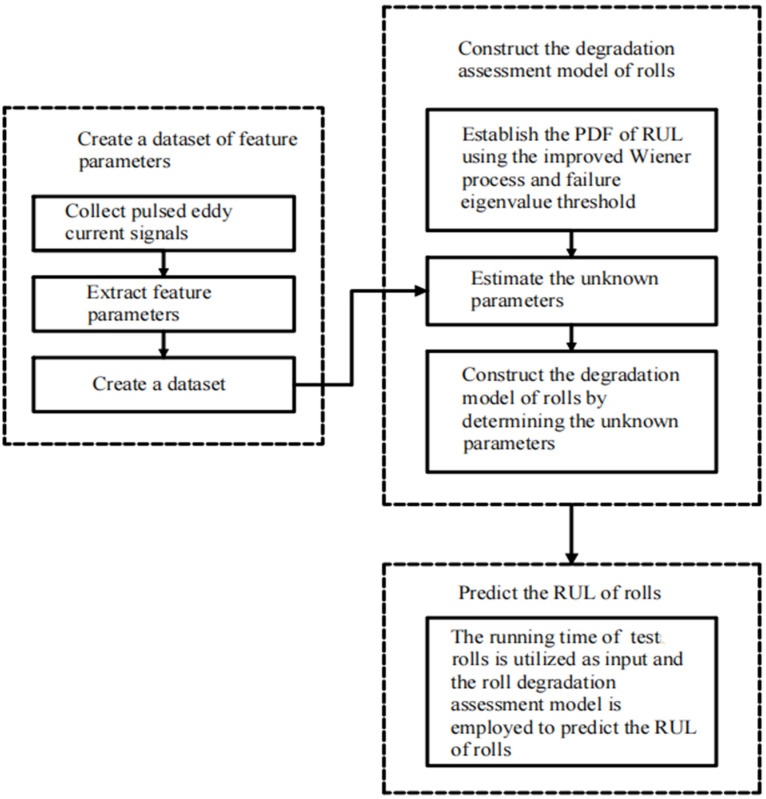
An overall graphical illustration of the proposed method.

This paper introduces a method to characterize the roll degradation state and accurately predict the RUL of hot rolling work rolls. It integrates pulsed eddy current testing with an improved Wiener process and comprises three parts including feature extraction, degradation model construction and unknown parameters estimation, and RUL prediction. An overall graphical illustration of the proposed method is shown in Figure 2. The detailed procedures are as follows:

1. Create a dataset of feature parameters

Collect the original pulsed eddy current signal from test roll surface and utilize a time–frequency domain analysis technique to extract the feature parameters that reflect the degradation process of the roll; create a feature parameters dataset by acquiring the feature parameters for both non-operating rolls and real-time operating rolls.

2. Construct the degradation assessment model of rolls

Introduce a kernel function into standard Wiener process to construct the improved Wiener process degradation model, determine the eigenvalue threshold of roll failure according to the current working state, and construct the PDF of RUL. Take the feature parameters of rolling time and its historical moments as the input and estimate the unknown parameters at the current moment. Input the calculated unknown parameters into the PDF of RUL to obtain the roll degradation assessment model.

3. Predict the RUL of rolls

Take the running time point of the test roll as input and use the roll degradation assessment model to calculate the RUL of the roll at each moment, thereby realizing the real-time adaptive RUL prediction.

### 3.2. Degradation Modeling Based on Improved Wiener Process

Suppose the degradation amount of the sample at time t is $X\left(t\right)$, then the one-dimensional linear Wiener process $X\left(t\right)$ (t≥0) can be defined as a stochastic process satisfying the following properties:

1. The increment at any time obeys a normal distribution $∆X=X\left(t+∆t\right)-X\left(t\right)\sim N\left(\eta ∆t{,\sigma }^{2}∆t\right)$, where η and σ represent the drift coefficient and diffusion coefficient, respectively;
2. The increments in any two disjoint time periods are independent of each other. That is, for any $0<t_{1}<\cdots <t_{n}$, $X\left(t_{1}\right)-X\left(t_{0}\right),X\left(t_{2}\right)-X\left(t_{1}\right),\cdots ,X\left(t_{n}\right)-X\left(t_{n-1}\right)$ are independent of each other;
3. $X\left(0\right)=0$, and it is continuous at t=0.

By analyzing the historical data of rolls, it can be found that the roll degradation path has obvious nonlinear characteristics. Therefore, the linear Wiener process makes it difficult to describe the dynamic characteristics of nonlinear degradation. The nonlinear Wiener process degradation model can be given by the following formula:

(5) $$X\left(t\right)=\eta \Lambda \left(t\right)+\sigma Β\left(t\right)$$

where $\Lambda \left(t\right)$ is the time scale conversion function, and σ is the diffusion coefficient used to adjust the diffusion degree of the Brownian motion function.

The degradation of rolls is a complex linear process with multiple factors coupled with each other. At present, most of the research based on the nonlinear Wiener process assumes that the time scale conversion function satisfies an exponential form, that is, $\Lambda \left(t\right)=t^{\lambda }$ [25]. However, the Wiener process based on the exponential function is only suitable for a small number of nonlinear degradation problems. Therefore, a more flexible drift function needs to be studied to improve the modeling ability for complex nonlinear problems.

Kernel functions can map data from the original space to a high-dimensional feature space, so that data that are non-linearly separable in the original space become linearly separable in the high-dimensional space, or it can allow the data to have better separability in the high-dimensional space [26]. In light of this idea, this paper introduces kernel functions into roll degradation modeling based on the Wiener process to better characterize the uncertainties inherent in the roll degradation process. First, sample differences are captured through the dynamic weighting of data points over time, allowing the model to reflect variations in initial conditions and degradation paths across different samples. Second, measurement errors are mitigated by the smoothing properties of kernel functions, which effectively reduce the impact of such errors and enhance the model’s predictive accuracy and robustness. Finally, environmental interference, such as fluctuations due temperature and load variations, is managed through the localized weighting of data points. This enables the model to adapt to nonlinear environmental changes, thereby accurately capturing their influence on the degradation process. The degradation model based on the improved Wiener process is given by the following formula:

(6) $$X\left(t\right)=\left(\sum_{i=1}^{m}w_{i}k\left(t,t_{i}\right)+w_{0}\right)+\sigma Β\left(t\right)$$

where $k\left(t,t_{i}\right)$ is the kernel function, $w_{i}$ is the weight coefficient, $w_{0}$ is the intercept, $t_{i}$ is the time of the i-th measurement, m is the total number of measurements, and $y\left(t|W\right)=\sum_{i=1}^{m}w_{i}k\left(t,t_{i}\right)+w_{0}$, $W={\left[w_{0}w_{1}\cdots w_{m}\right]}^{T}$.

In Formula (6), $k\left(t,t_{i}\right)$ is the general form of the kernel function. The choice of kernel function determines the model’s ability to handle nonlinear problems and affects the accuracy and effectiveness of the degradation model in predicting roll performance. The commonly used kernel functions include linear kernel function, polynomial kernel function, Gaussian kernel function, Laplace kernel function, Sigmoid kernel function, etc. In practical applications, according to the prediction effect of the measured roll data, a suitable kernel function can be selected and embedded into the drift function term of the model.

Based on Formula (6), the independent increment of the improved Wiener process degradation model is $X={\left[∆x_{1}\;∆x_{2\;}\cdots ∆x_{n\;}\right]}^{T}$, and its joint probability density function (PDF) obeys a multivariate Gaussian distribution:

(7) $$p\left(x|w,\sigma \right)=\frac{1}{\sqrt{{\left(2\pi \right)}^{n}|E|}}\times exp\left[-\frac{1}{2}{\left(x-{\left[\varphi \left(∆x_{1}\right)\cdots \varphi \left(∆x_{n}\right)\right]}^{T}\times w\right)}^{T}E^{-1}\left(x-{\left[\varphi \left(∆x_{1}\right)\cdots \varphi \left(∆x_{n}\right)\right]}^{T}w\right)\right]$$

where $\varphi \left(∆x_{i}\right)=\left[1,k\left(∆x_{i},∆x_{1}\right)\;,\cdots \;,k\left(∆x_{i},∆x_{n}\right)\right]$, and E is the prior covariance matrix of elements $E\left(i,j\right)=\sigma^{2}min\left(t_{i,}t_{j,}\right)$. The values of the unknown parameters w and σ need to be estimated according to the roll degradation historical data.

### 3.3. Unknown Parameters Estimation

Estimate the unknown parameters of the model described in Section 3.1. Firstly, the posterior distribution of parameters w and σ is derived based on Bayesian theory to obtain the Marginal Likelihood Function (MLF). Then, by maximizing the MLF, the Maximum Likelihood Estimation (MLE) of parameters w and σ can be obtained. However, overfitting may occur when using MLE to estimate parameters [27]. To avoid the occurrence of overfitting, the prior distribution of parameters, w, can be defined as a Gaussian distribution with a mean equal to 0, that is:

(8) $$p\left(w|\alpha \right)=\prod_{i=0}^{m}\left(\frac{\alpha_{i}}{\sqrt{2\pi }}exp\left(\frac{-\alpha_{i}w_{i}^{2}}{2}\right)\right)$$

where $\alpha =\left[\alpha_{0}{,\;\;\alpha }_{1},\cdots ,\;\alpha_{m}\right]$ is the hyperparameter vector of w.

According to multilayer Bayesian prior distribution, the hyper-prior distributions of α and β are defined to follow Gamma distributions, that is:

(9) $$p\left(\alpha \right)=\prod_{i=0}^{m}Gamma\left(\alpha_{i\;}|a,b\right)$$

(10) $$p\left(\beta \right)=Gamma\left(\beta |c,d\right)$$

where $\beta =\sigma^{-2}$, $Gamma\left(\alpha_{i\;}|a,b\right)=\Gamma \;{\left(a\right)}^{-1}b^{a}\alpha^{a-1}e^{-ba}$, $\Gamma \;\left(a\right)=\int_{0}^{\infty }t^{a-1}e^{-t}dt$. When choosing an uninformative prior as the hyper-prior distribution of α and $\sigma^{2}$, the parameters should be small enough, such as $a=b=c=d=10^{-4}$.

Based on the Bayesian formula, the posterior distributions of unknown parameters w, α and σ can be expressed as follows:

(11) $$p\left(w,\alpha ,\sigma^{2}|x\right)=p\left(w|x,\alpha ,\sigma^{2}\right)p\left(\alpha ,\sigma^{2}|x\right)$$

According to Bayesian theorem, the calculation formula of w is as follows:

(12) $$p\left(w|x,\alpha ,\sigma^{2}\right)=\frac{p\left(x|w,\sigma^{2}\right)p\left(w|\alpha \right)}{p\left(x|\alpha ,\sigma^{2}\right)}={\left(2\pi \right)}^{-\left(m+1\right)/2}{\left|\;E\;\right|}^{-1/2}\times exp\left[-\frac{1}{2}{\left(w-\mu \right)}^{T}E^{-1}\left(w-\mu \right)\right]$$

The estimation values of parameters α and σ can be obtained by maximizing the MLF $p\left(\alpha ,\sigma^{2}|\;x\right)$, that is, maximizing $p\left(x|\alpha ,\sigma^{2}\right)$:

(13) $$p\left(\;x|\alpha ,\sigma^{2}\right)=\int p\left(\;x|w,\sigma^{2}\right)p\left(\;w|\alpha \right)dw={\left(2\pi \right)}^{-\frac{m}{2}}\sigma^{2}+\Phi A^{-1}\Phi^{T}{|}^{-\frac{1}{2}}\times exp\left[-\frac{1}{2}x^{T}{\left(\sigma^{2}\Phi A^{-1}\Phi^{T}\right)}^{-1}x\right]$$

where $\Phi ={\left[\varphi \left(∆x_{1}\right)\varphi \left(∆x_{2}\right)\cdots \varphi \left(∆x_{n}\right)\right]}^{T}$, and $A=diag\left(\alpha_{1},\cdots \;,\alpha_{m}\right)$, the posterior covariance matrix and the mean of Equation (13) are:

(14) $$C={\left(\sigma^{-2}\Phi^{T}\Phi +diag\left(\alpha_{1},\cdots \;,\alpha_{m}\right)\right)}^{-1}$$

(15) $$\mu ={\left(diag\left(\alpha_{1},\cdots \;,\alpha_{m}\right)+\Phi^{T}E^{-1}\Phi \right)}^{-1}\Phi^{T}E^{-1}x$$

Design an iterative estimation algorithm to obtain the approximate solutions of hyperparameters α and $\sigma^{2}$ in Equation (13). According to the Mackay method [28], let Equation (12) be equal to 0, and after sorting, the iterative updated values of α and $\sigma^{2}$ are as follows:

(16) $$\alpha_{i}^{new}=\gamma_{i}/\mu_{i}^{2}$$

(17) $${\left(\sigma^{2}\right)}^{new}=\frac{{\left\|x-\Phi \mu \right\|}^{2}}{m}-E_{ii}\gamma_{i}$$

where $\mu_{i}$ is the i-th posterior mean weight of Equation (15), $\gamma_{i}\equiv 1-\alpha_{i}E_{ii}$, and $E_{ii}$ is the i-th diagonal element of the posterior variance matrix of Equation (14), which can be calculated from the values of α and $\sigma^{2}$. In the iteration, if $\alpha_{i}$ is very large and $w_{i}$ is strictly constrained by its prior probability, then let $E_{ii}=\alpha_{i}^{-1},\;\gamma_{i}\approx 0$; if $\alpha_{i}$ is very small and $w_{i}$ adapts to the data, let $\gamma_{i}\approx 1$.

For a single set of roll degradation vector sample x, in order to obtain the corresponding predicted value $x_{*}$, based on probability theory and Bayesian theorem, the following formula can be obtained:

(18) $$p\left(x_{*}|x\right)=\int p\left(x_{*}|w,\sigma^{2}\right)p\left(w,\sigma^{2}|x\right)dwd\sigma^{2}=\int p\left(x_{*}|w,\alpha ,\sigma^{2}\right)p\left(w,\alpha ,\sigma^{2}|x\right)dwd\alpha d\sigma^{2}$$

Substituting Equation (11) into Equation (18), the conditional probability of the predicted value $x_{*}$ can be obtained as follows:

(19) $$p\left(x_{*}|x\right)=\int p\left(x_{*}|w,\alpha ,\sigma^{2}\right)p\left(w,\alpha ,\sigma^{2}|x\right)dwd\alpha d\sigma^{2}$$

According to Bayesian theorem, the posterior probability $p\left(w,\alpha ,\sigma^{2}|x\right)$ in Equation (19) is decomposed into $p\left(w|x,\alpha ,\sigma^{2}\right)p\left(\alpha ,\sigma^{2}|x\right)$, that is:

(20) $$p\left(x_{*}|x\right)=\int p\left(x_{*}|w,\alpha ,\sigma^{2}\right)p\left(w|x,\alpha ,\sigma^{2}\right)\times p\left(\alpha ,\sigma^{2}|x\right)dwd\alpha d\sigma^{2}$$

Update the parameters based on Equations (16) and (17). Calculate the Equations (14) and (15) at the same time, updating the posterior statistics C and μ, until the maximum number of cycles is reached or the gradient of output result is less than the convergence condition (i.e., $10^{-3}$). Since there is a tendency for some values to approach infinity during the iteration process, according to the properties of the kernel function, delete the weights and kernel functions corresponding to all $\alpha_{i}$ in $\alpha_{i}>\alpha_{max}$ in the iteration process and set $\alpha_{max}=10^{5}$. In this way, the sparsity of the improved degradation model can be achieved.

Denote $\alpha_{MP}$ and $\sigma_{MP}^{2}$ as the optimal values after stopping iteration. At this time, given a new set of roll degradation data, its predicted output can be obtained by the following formula:

(21) $$p\left(x_{*}|x,\alpha_{MP},\sigma_{MP}^{2}\right)=\int p\left(x_{*}|w,\sigma_{MP}^{2}\right)p\left(w|x,\alpha_{MP},\sigma_{MP}^{2}\right)dw$$

Since the two integral terms on the right side of the equation both satisfy Gaussian distribution, the predicted value $x_{*}$ satisfies the following distribution:

(22) $$p\left(x_{*}|x,\alpha_{MP},\sigma_{MP}^{2}\right)=N\left(x_{*}|y_{*\;},\sigma_{*}^{2}\right)$$

where the mean $y_{*\;}$ and variance $\sigma_{*}^{2}$ of the predicted output $x_{*}$ are:

(23) $$\begin{array}{c} y_{*\;}=\mu^{T}\varphi \left(x_{*}\right)\\ \sigma_{*}^{2}=\sigma_{mp}^{2}+{\varphi \left(x_{*}\right)}^{T}E\varphi \left(x_{*}\right) \end{array}$$

From the above formula, it can be seen that the prediction error of the improved model consists of two parts: the inherent noise of the data and the uncertainty in the process of model weight estimation. The improved model can better avoid overfitting by constraining parameter w and adjusting the diffusion coefficient $\sigma Β\left(t\right)$.

### 3.4. RUL Prediction

Considering the failure characteristic value threshold ξ of the roll, the degradation trajectory of the workable rotation amount of the roll is described by the improved Wiener process degradation model. Then, the useful life T of the roll is the time when the performance degradation amount $X\left(t\right)$ of the roll first reaches the threshold:

(24) $$T=inf\left\{T\;|\;X\left(t\right)\geq \xi ,t>0\right\}$$

where $inf\left\{\cdot \right\}$ represents the infimum of a function. Then, the RUL of the roll at any time $t_{k}$ is defined as follows:

(25) $$H_{k}=inf\left\{h_{k};X\left(t_{k}+h_{k}\right)\geq \xi |X\left(t_{k}\right)<\xi \right\}$$

The first passage time of the improved Wiener process obeys the inverse Gaussian distribution. In order to estimate the probability density of the first passage time of the roll, based on Formulas (7) and (25), the PDF of RUL can be derived as:

(26) $$f_{2}\left(t\;|\;w\right)=\frac{\xi }{\sqrt{2\pi \sigma^{2}t^{3}}}exp\left[-\frac{{\left(\xi -y\left(t\;|\;w\right)\right)}^{2}}{{2\sigma }^{2}t}\right]\frac{dy\left(t\;|\;w\right)}{dt}$$

According to the feature parameters dataset, taking the feature parameters at a certain moment and their historical moments as inputs allows to obtain the unknown parameters at time. Substituting the obtained unknown parameters into the PDF of RUL, the solved PDF of RUL is obtained, which is the roll degradation assessment model. Taking the operation time points of the roll as inputs and using the roll degradation assessment model to calculate the RUL of rolls at each moment can realize the real-time RUL prediction of rolls.

## 4. Experimental Plan

### 4.1. Feature Extraction

**Figure 3:**
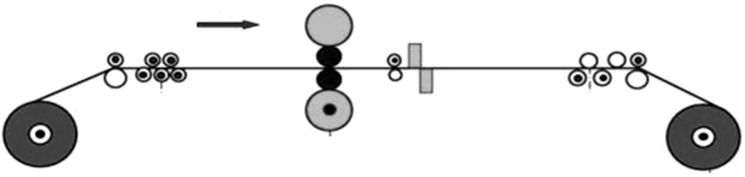
The schematic diagram of 2250 mm hot rolling mill leveling unit (single stand).

**Figure 4:**
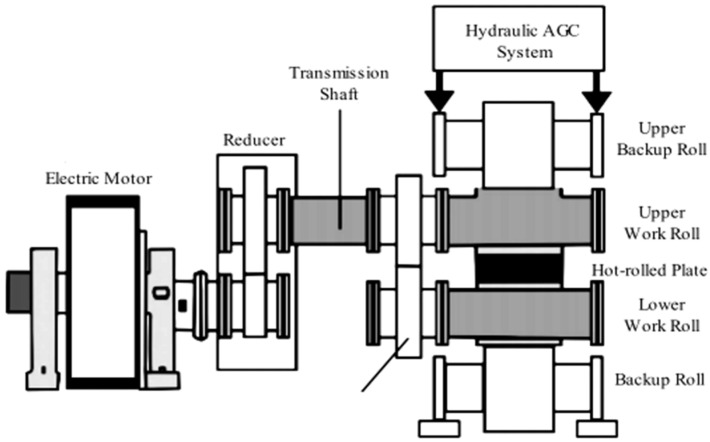
The schematic diagram of stands and rolls.

**Figure 5:**
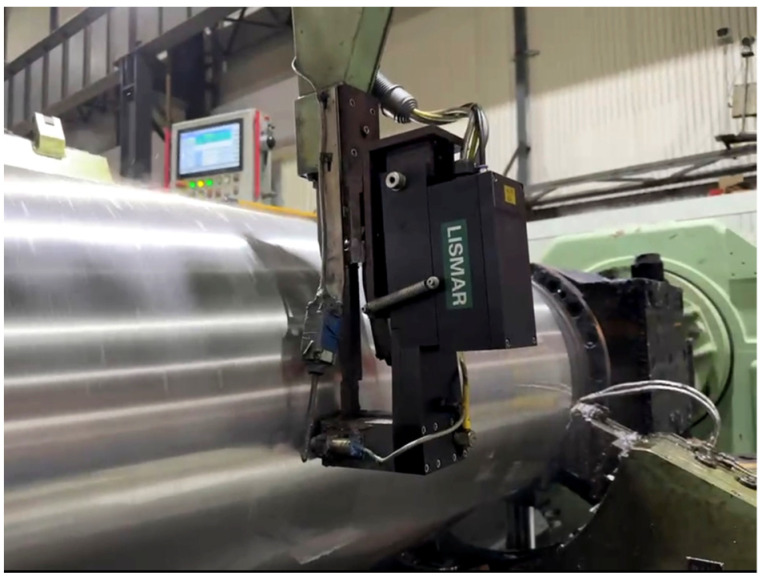
The LISMAR DATAMES detection platform on site.

The data in this section are derived from a 2250 mm hot rolling mill leveling unit in a Chinese steel firm. The mill leveling unit is a single-stand mill unit with a pair of work rolls. The schematic diagram of 2250 mm hot rolling mill leveling unit is shown in Figure 3, and the schematic diagram of the stands and rolls is given in Figure 4. At present, the production line has implemented a pulsed eddy current online detection system called LISMAR for the condition monitoring. It is an automatic detection system and performs detection throughout the entire process, starting from zero revolutions after grinding and continuing until the operation reaches the threshold. Combined with the LISMAR DATAMES detection platform, it can effortlessly acquire and retrieve the eddy current information data from the roll surface. The LISMAR DATAMES detection platform on site is shown in Figure 5.

The first set of analyses examined the impact of different frequencies on the pulsed eddy current signal of roll surface. By taking roll No. T4-W418 as an example, Table 1 shows the original pulsed eddy current signal of the roll surface at zero revolutions in the frequency band from 0 to 20,000 Hz.

**Figure 6:**
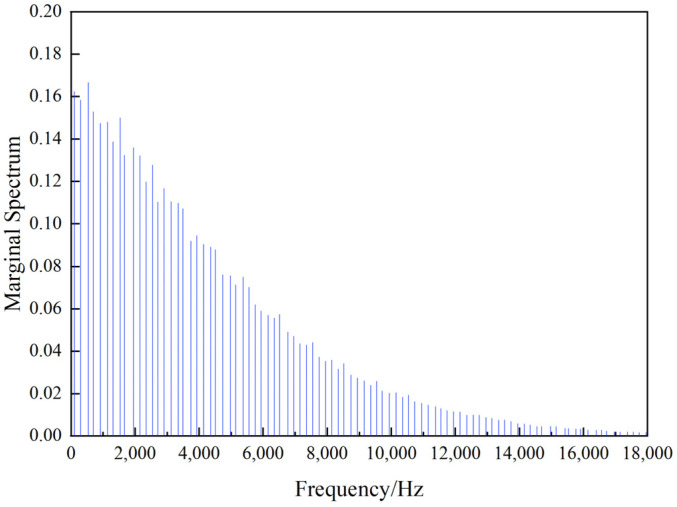
The marginal spectrum of the test roll (No. T4-W418).

According to the VMD-Hilbert method in Section 2, feature extraction is carried out on the original signal of the roll surface. Figure 6 gives the marginal spectrum of the test roll, which represents the energy distribution of each frequency sub-band of the signal.

As shown in Figure 6, the energy of the pulsed eddy current signal decreases from low frequency to high frequency and is mainly distributed in the frequency sub-bands from 0 to 18,000 Hz. The signal energy in the frequency sub-bands exceeding 18,000 Hz is very low. This may be due to the fact that the signal in the high frequency range is easily affected by the oxide layer on the surface of rolls. At the same time, industrial noises are inevitably present during the operation, and these noises mainly act on high frequency ranges. Therefore, signals in frequency bands from 0 to 18,000 Hz are relatively effective to represent the degradation state of work rolls.

**Figure 7:**
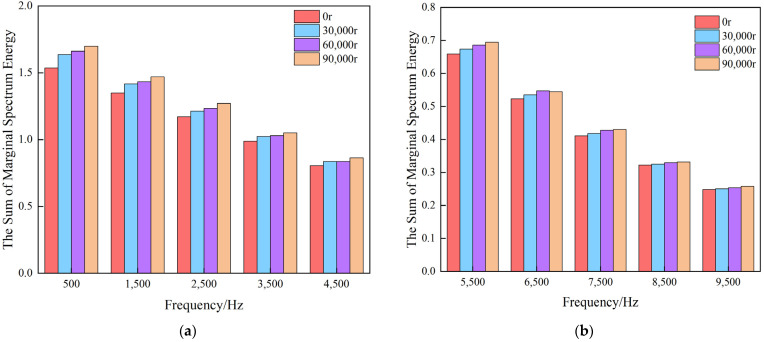

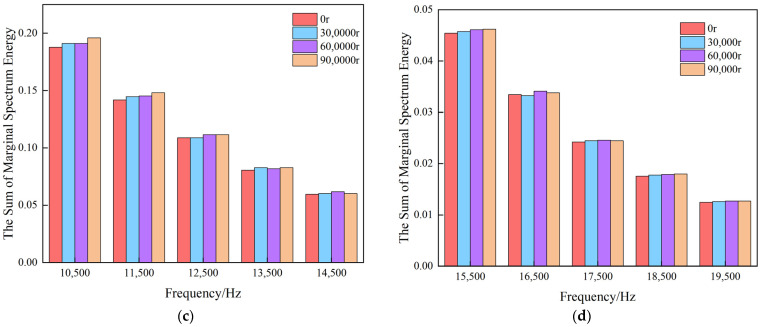
The sum of marginal spectrum energy in each frequency sub-band: (**a**) 0–4500 Hz; (**b**) 5500–9500 Hz; (**c**) 10,500–14,500 Hz; (**d**) 15,500–19,500 Hz.

Figure 7 presents the sum of marginal spectrum energy in each frequency sub-band (0–19,500 Hz) at different time-points (0–90,000 r). It clearly demonstrates that the roll data at various time periods within the frequency bands from 0 to 4500 Hz exhibit significant differences and good distinguishability. As the frequency increases, these differences gradually decrease, and cross-over phenomena even occur. Therefore, the marginal spectrum energy in the frequency sub-bands from 0 to 4500 Hz is extracted in this paper as the effective feature to represent the degradation state of work rolls. The marginal spectrum energy can be calculated as follows:

(27) $$MSE=\int_{0}^{4500}h\left(\omega \right)d\omega$$

**Figure 8:**
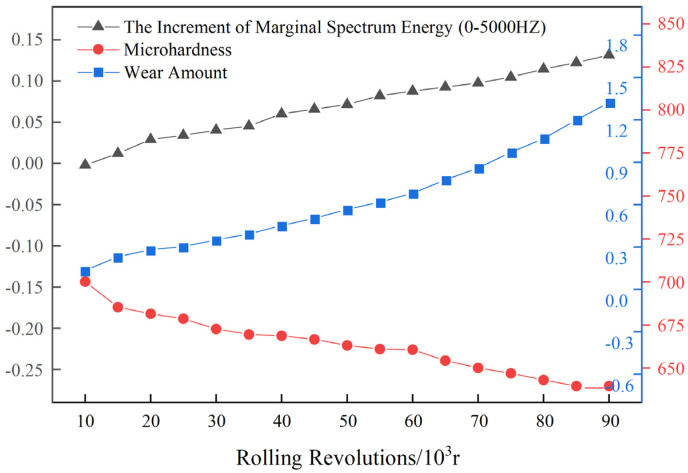
The changes in microhardness of high-speed steel cladding layer, wear amount, and increment of marginal spectrum energy.

To minimize the variations among individuals, the increment of marginal spectrum energy is utilized as the input for RUL prediction of work rolls. Figure 8 illustrates the changes in the microhardness of high-speed steel cladding layer, wear amount, and the increment of marginal spectrum energy.

It can be seen from the above figure that there is a negative correlation between the microhardness and the increment of marginal spectrum energy, and its correlation coefficient is about −0.97%. Conversely, there is a positive correlation between the wear amount and the increment of marginal spectrum energy, and its correlation coefficient is about 0.91%. Thus, the increment of marginal spectrum energy can effectively describe the roll degradation state.

### 4.2. Kernel Function Selection

The selection of kernel function directly affects the prediction accuracy of the improved degradation model, as well as the model sparsity and training time. Based on the experimental data in the previous section (the roll No. T4-W418 on the 2250 single-stand temper mill unit), according to different kernel functions, five groups of experiments are conducted for comparison. The Root Mean Square Error (RMSE), Goodness of Fit (CD), and Mean Absolute Error (MAE) are used to evaluate the algorithm performance [29]. The results are shown in Table 2. Smaller values of RMSE and MAE indicate higher algorithm prediction accuracy. The closer the CD value is to 1, the better the prediction model. The RMSE, CD, and MAE are calculated, respectively, as follows:

(28) $$\alpha_{RMSE}=\sqrt{\frac{1}{n^{\prime }}\sum_{t=1}^{n^{\prime }}{\left(x\left(t\right)-\hat{x}\left(t\right)\right)}^{2}}$$

(29) $$\eta_{CD}=\frac{\sum_{t=1}^{n^{\prime }}{\left(\;\hat{x}\left(t\right)-\bar{x}\right)}^{2}}{\sum_{t=1}^{n^{\prime }}{\left(\;x\left(t\right)-\bar{x}\right)}^{2}}$$

(30) $$\alpha_{MAE}=\frac{1}{n^{\prime }}\sum_{t=1}^{n^{\prime }}|\;x\left(t\right)-\hat{x}\left(t\right)\;|$$

It can be seen from the above table that the RMSE, CD, and MAE of the Gaussian kernel function are all superior to other kernel functions. This indicates that the original data have better linear features after being mapped to the high-dimensional feature space through the Gaussian kernel function. It also proves that different kernel functions have a large performance difference in different application scenarios. At the same time, the improved Wiener process degradation model based on the Gaussian kernel function has better sparsity, and it is more convenient to realize online monitoring. Therefore, under the experimental conditions in this paper, introducing the Gaussian kernel function into standard Wiener process can obtain better experimental results.

## 5. Results and Discussion

**Figure 9:**
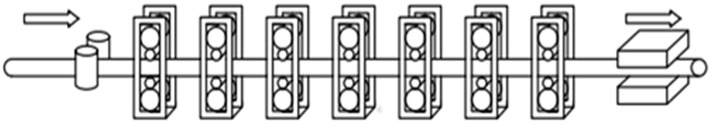
The schematic diagram of 2250mm hot rolling mill unit (seven stands).

The data in this section are derived from all seven stands of the 2250 mm hot rolling production line. The signal data are firstly sorted and cleaned. Then, after considering the factors such as data integrity, the rolls No. T4-W311, No. T4-W421, and No. T4-X303 are taken as examples for comparison to verify the practicability and effectiveness of the proposed degradation model and RUL prediction method in this paper. The schematic diagram of the seven-stands hot rolling mill unit is shown in Figure 9. Examples of the original signals in frequency sub-bands from 0 to 4500 Hz are shown in Table 3.

The proposed improved method is used to predict the RUL of rolls No. T4-W311, No. T4-W421, and No. T4-X303 on stands F7, F7, and F5. Parameter estimation is conducted using the Bayesian framework, as detailed in Section 3.3, with Matlab utilized for the implementation. The estimation process involves the following key steps:

Step 1: Initialization. The initial values for the diffusion parameter $\sigma^{2}$ and the kernel function parameter α are set. The posterior covariance matrix C and mean μ are also initialized.

Step 2: Bayesian Inference. The posterior distribution of the model parameters is derived based on Bayesian theory. The marginal likelihood function is formulated to estimate the posterior covariance and mean of the kernel function weights.

Step 3: Iterative Parameter Update. An iterative algorithm is employed to update the parameters. First, the posterior covariance C and mean μ are computed using Equations (14) and (15). The kernel parameter α is updated according to Equation (16), and the diffusion parameter $\sigma^{2}$ is updated using Equation (17). During each iteration, parameters α exceeding a predefined threshold are removed to achieve model sparsity, improve generalization, and avoid overfitting.

Step 4: Convergence. The iterative process continues until either the maximum number of iterations is reached or the change in parameter values (gradient) falls below a predefined threshold.

**Figure 10:**
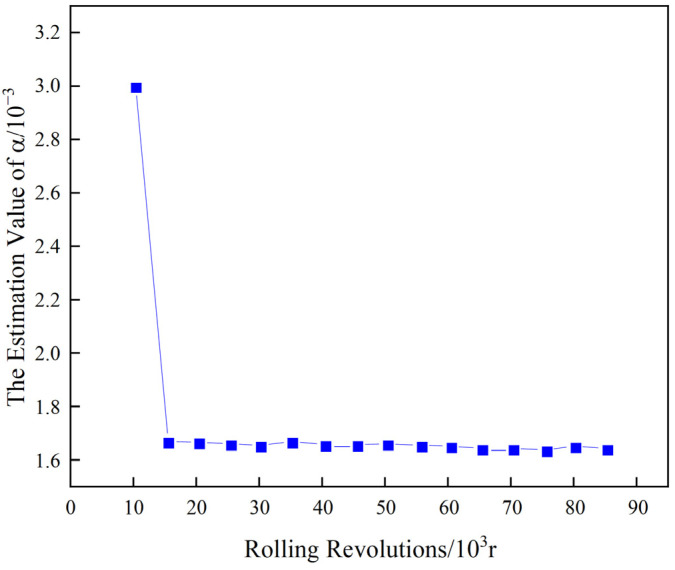
The final value of unknown parameter α.

**Figure 11:**
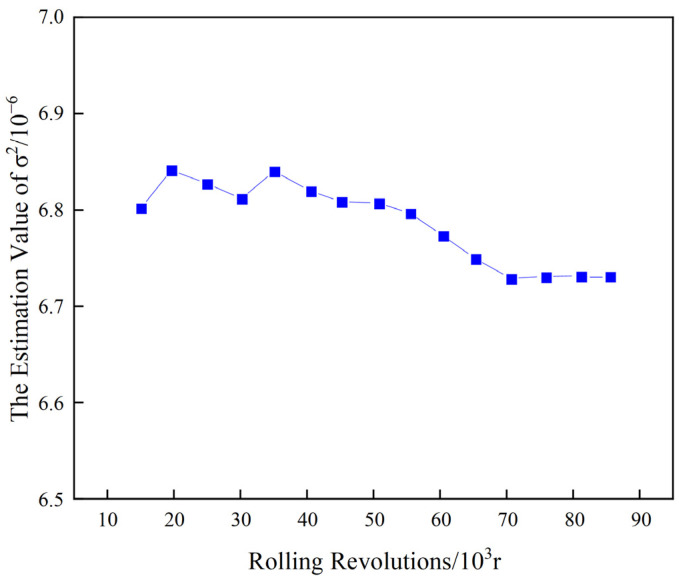
The final value of unknown parameter $\sigma^{2}$.

Taking roll No. T4-W311 as an example, the experiment is conducted with 10 sample groups. Since the hot rolling work rolls operate under the same pass, the degradation trends of these 10 samples are similar. Therefore, samples 1–9 are used as historical monitoring data, while sample 10 is used as real-time monitoring data for the current operation. For the historical data, the full-cycle data comprising 90,000 cycles are used to estimate the unknown parameters. At $t=4\times 10^{4}$ revolutions, the initial threshold ξ is set to 1, with initial parameters α=0.2 and $\sigma^{2}=0.001$. After 100 iterations of Bayesian updates, the final converged values of α and $\sigma^{2}$ are obtained, as shown in Figure 10 and Figure 11.

As can be seen from Figure 10 and Figure 11, after 100 iterations of Bayesian updates, α and $\sigma^{2}$ converged, resulting in the final values of $\alpha =1.66\times 10^{-3}$ and $\sigma^{2}=6.78\times 10^{-6}$. Substituting these into Equation (26), the RUL of the roll No. T4-W311 at $t=4\times 10^{4}$ is calculated to be $0.9634\times 10^{4}$ revolutions. Therefore, the predicted RUL is 49,643.10 revolutions. Similarly, the RUL for roll No. T4-W421 at $t=3\times 10^{4}$ and roll No. T4-X303 at $t=2\times 10^{4}$ can be calculated, as presented in Table 4.

**Figure 12:**
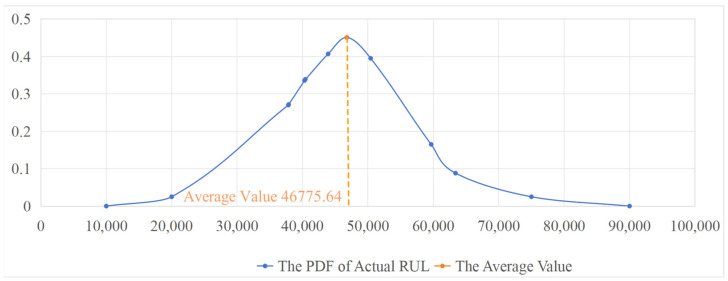
The PDF of actual RUL of roll T4-W311 on stand F7.

**Figure 13:**
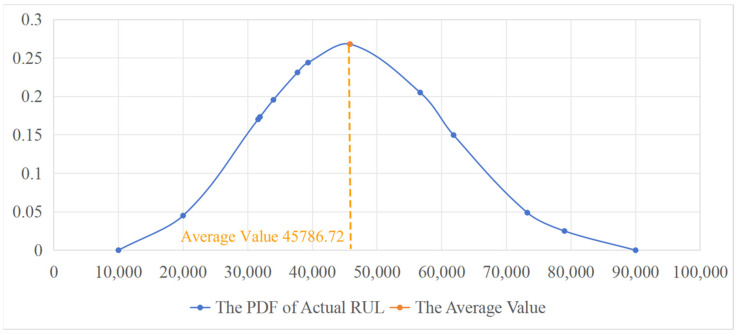
The PDF of actual RUL of roll T4-W421 on stand F7.

**Figure 14:**
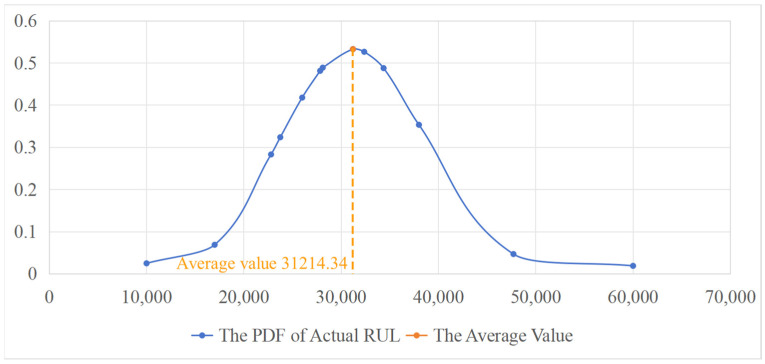
The PDF of actual RUL of roll T4-X303 on stand F5.

The actual in-service operation, rolling kilometerage, and converted in-service RUL of each roll are presented in Table 5, Table 6 and Table 7, respectively. The actual RUL is calculated using the Equation (31). The PDFs of the actual RUL are shown in Figure 12, Figure 13 and Figure 14, with the mean value of each PDF taken as the actual average RUL (in revolutions).

(31) $$RUL=\frac{Rolling\;kilometerage\times 10^{6}}{\pi \times Diameter}$$

To verify the effectiveness of the proposed method in this paper, the RUL prediction results are compared with the actual average RUL. The performance of the method is evaluated by the error between the predicted value and the actual value. The results are shown in Table 8.

As can be seen from the above table, the predicted RUL based on improved Wiener process proposed in this paper is relatively close to the actual value. The predicted RUL value is slightly larger than the actual RUL. This is because during the simultaneous operation of all seven stands, if the eigenvalue threshold of the roll surface at any stand is abnormal, the rolls of the other remaining stands need to be replaced synchronously, thus causing a certain waste to their RUL. The predicted average error is about 5.66%, which can meet the actual prediction needs on site. Therefore, the combination of the standard Wiener process and the Gaussian kernel function can effectively predict the RUL of rolls and has good model versatility and practicability. The proposed method has a strong guiding significance for the roll change decision on the production site.

**Figure 15:**
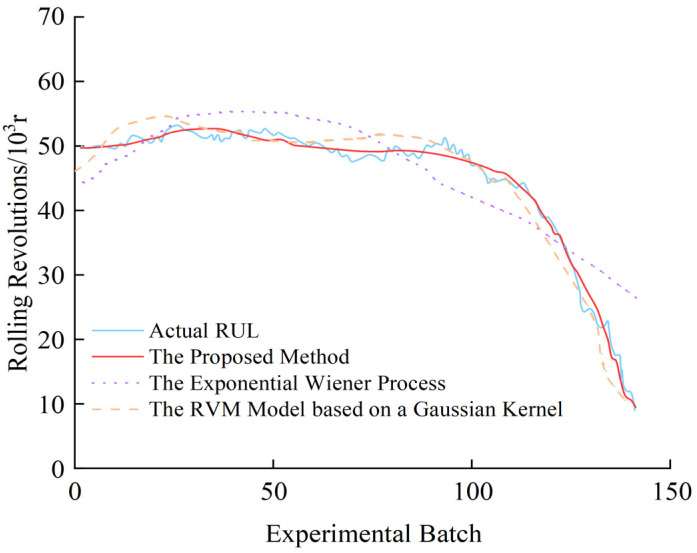
Comparison of the modeling performance of the proposed method (roll No. T4-W418).

The experiment further compares the performance of the proposed model with that of the exponential Wiener process model and the Relevance Vector Machine (RVM) model using a Gaussian kernel for degradation modeling of the nonlinear time series of roll No. T4-W418, as shown in Figure 15.

As illustrated in the figure, the exponential Wiener process shows a significant deviation between its predicted and actual RUL values, indicating poor adaptability in modeling roll degradation. This limitation primarily arises from the simplicity of the exponential function, which restricts its ability to capture the complex nonlinear characteristics of the roll’s actual degradation path. In contrast, the RVM model with a Gaussian kernel can capture more intricate nonlinear degradation paths, resulting in improved prediction performance. However, it demonstrates limited robustness in roll degradation modeling. Given the complexity of key equipment structures and the strong random disturbances present in the steel hot rolling production line, accurately characterizing the stochastic nature of equipment performance evolution is crucial. The RVM model with a Gaussian kernel struggles to handle the randomness present in the degradation path, making it challenging to represent the true degradation trajectory of the roll. The proposed model, however, effectively predicts the degradation trend of the hot roll, with predicted values generally falling within the acceptable error range. Its prediction performance not only significantly surpasses that of the exponential Wiener process but also shows a marked improvement over the RVM model with a Gaussian kernel.

The RMSE, CD, and MAE of these three models are calculated for a quantitative analysis. The results are presented in Table 9. From Table 9, it can be seen that the proposed method exhibits a significant improvement over both the exponential Wiener process and the RVM model with a Gaussian kernel. Specifically, it achieves a reduction in RMSE by approximately 85.47% and 41.20% compared to the exponential Wiener process and the RVM model, respectively. Furthermore, the CD is enhanced by 121% relative to the exponential Wiener process and by 19.76% compared to the RVM model, indicating a considerably better model fit (closer to value 1). In terms of MAE, the proposed method achieves reductions of 85.66% and 42.61% compared to the exponential Wiener process and the RVM model, respectively. These results clearly demonstrate the superior prediction accuracy and robustness of the proposed method.

**Figure 16:**
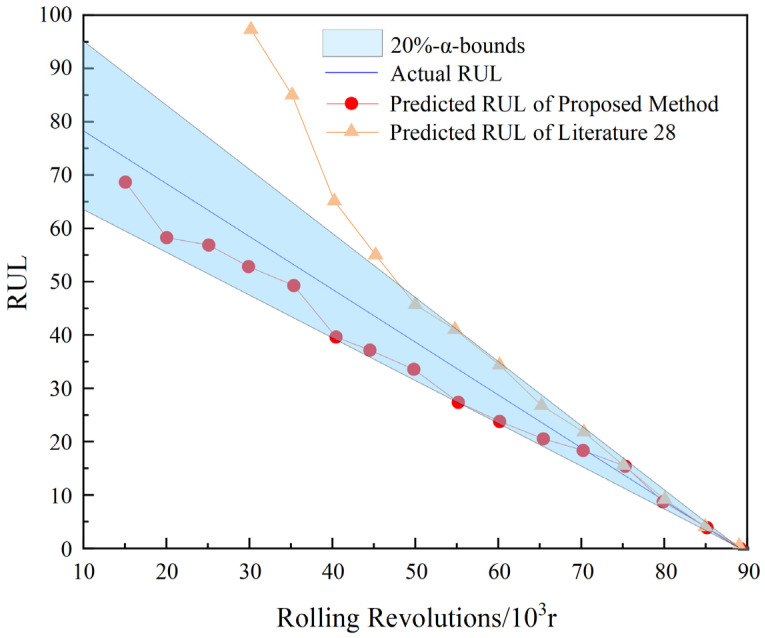
The comparison of RUL prediction results (roll No. T4-X303).

Furthermore, in order to verify the superiority of proposed method, the expectation principle is used to fit the RUL prediction trajectories of different methods. The blue area represents the 20% confidence interval of the RUL prediction, and the abscissa is the operating time. The more parts of RUL prediction trajectory fall within the confidence interval, the more accurate the prediction result. Taking roll No. T4-X303 as an example, the prediction results of the method in the literature [30] and the proposed method in this paper are analyzed and compared, as shown in Figure 16. As can be seen from the figure, the RUL trajectory corresponding to the proposed method is basically all within the confidence interval. Meanwhile, for the method in the literature [30], when the roll operating time is less, its predicted RUL has a large deviation. This shows that the proposed method has strong prediction accuracy throughout the detection process.

## 6. Conclusions

This paper proposes a degradation modeling and RUL prediction method to address the inherent randomness and nonlinearity in the roll degradation process. The VMD-Hilbert method is employed to extract roll surface features, with the increment of marginal spectrum energy serving as a quantitative indicator of the degradation state. To capture the nonlinear degradation paths of work rolls, a Gaussian kernel function is integrated into the standard Wiener process, and unknown parameters are estimated using a Bayesian framework. An eigenvalue threshold is defined to integrate both historical and real-time data, constructing a comprehensive degradation assessment model and facilitating accurate RUL prediction.

The proposed method is validated through its application in a real-world case study from a Chinese firm, demonstrating its suitability for practical RUL prediction requirements in industrial settings. When compared to traditional approaches, such as the exponential Wiener process and the RVM model with a Gaussian kernel, the proposed method shows significant improvements in predictive accuracy, model fit, and robustness. Comparative analysis with other algorithms from the related literature further underscores its advantages, as it achieves a higher prediction accuracy throughout the monitoring process, with most RUL predictions falling within an acceptable confidence interval. These findings highlight the method’s robustness, versatility, and clear superiority in both prediction accuracy and model stability, making it a valuable tool for roll degradation monitoring in industrial applications.

However, due to the vast range of product types in the steel rolling production line, the proposed method does not consider specific details such as roll scheduling and roll material. Additionally, the various phases of degradation are not incorporated into the degradation modeling due to risk control requirements. Furthermore, this paper examines roll degradation status solely by analyzing pulsed eddy current signals, suggesting that future work could combine several non-destructive testing technologies to improve the accuracy of RUL prediction.

## Figures and Tables

**Table 1:** Original pulsed eddy current signal (0 r) for band 0–20,000 Hz.

No	Frequency	The Original Signal
1	0–2250 Hz	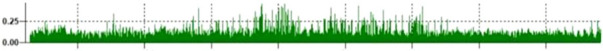
2	2500–4750 Hz	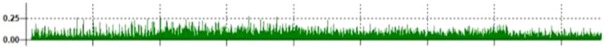
3	5000–7250 Hz	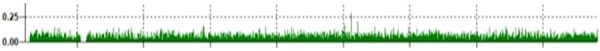
4	7500–9750 Hz	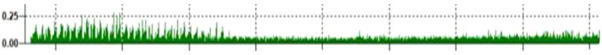
5	10,000–12,250 Hz	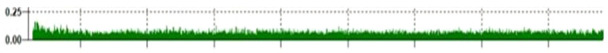
6	12,500–14,750 Hz	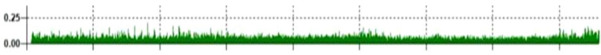
7	15,000–17,250 Hz	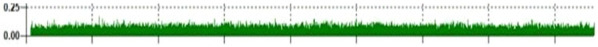
8	17,500–20,000 Hz	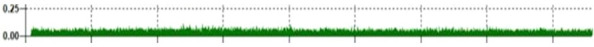

**Table 2:** The prediction results evaluation of different kernel functions.

Kernel Function	RMSE	CD	MAE
Gaussian kernel function	1054.31	0.94	821.85
Polynomial kernel function	7372.45	0.45	5621.06
Linear kernel function	10,181.25	0	7209.84
Laplace kernel function	1961.28	0.82	1401.63
Sigmoid kernel function	10,181.15	0	7209.84

**Table 3:** The original pulsed eddy current signal in frequency sub-bands from 0 to 4500 Hz.

No	Roll/Stand	The Original Signal
1	T4-W311/F7	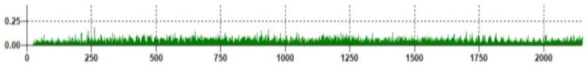 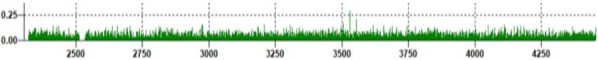
2	T4-W421/F7	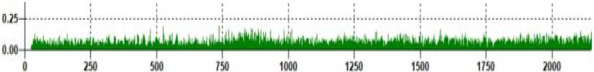 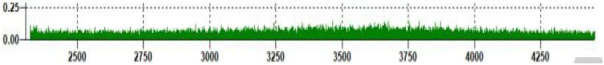
3	T4-X303/F5	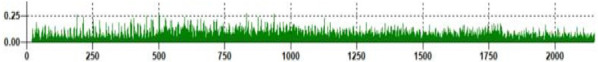 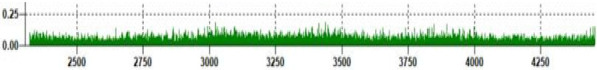

**Table 4:** The RUL prediction results of proposed method.

No	Roll	Stand	RUL (Revolutions)
1	T4-W311	F7	49,634.10
2	T4-W421	F7	48,245.54
3	T4-X303	F5	33,246.81

**Table 5:** The original data of roll T4-W311 on stand F7.

No	Roll	Time on Machine	Time off Machine	Diameter	Rolling Kilometerage	RUL
F7	T4-W311	2024-08-07 T01:18	2024-08-07 T06:02	695.570	82.752	37,888.55
F7	T4-W311	2024-08-06 T13:01	2024-08-06 T15:32	695.900	88.372	40,442.52
F7	T4-W311	2024-08-06 T00:57	2024-08-06 T03:37	696.280	130.462	59,671.98
F7	T4-W311	2024-08-05 T12:25	2024-08-05 T16:37	696.860	138.712	63,392.64
F7	T4-W311	2024-08-04T23:26	2024-08-05 T02:02	697.540	82.870	37,835.42
F7	T4-W311	2024-08-04T09:52	2024-08-04T11:52	697.760	96.234	43,923.08
F7	T4-W311	2024-08-02 T23:33	2024-08-03 T04:32	698.900	110.642	50,416.81
F7	T4-W311	2024-08-02 T12:32	2024-08-02 T15:32	699.360	102.770	46,798.93
F7	T4-W311	2024-08-01 T20:14	2024-08-02 T01:07	699.760	89.232	40,610.83

**Table 6:** The original data of roll T4-W421 on stand F7.

No	Roll	Time on Machine	Time off Machine	Diameter	Rolling Kilometerage	RUL
F7	T4-W421	2024-07-12 T06:35	2024-07-12 T09:32	642.820	92.476	45,815.24
F7	T4-W421	2024-07-10 T11:37	2024-07-10 T14:27	649.180	76.84	37,695.77
F7	T4-W421	2024-07-09 T19:04	2024-07-09 T21:12	649.410	65.028	31,889.80
F7	T4-W421	2024-07-08 T22:00	2024-07-08 T23:27	649.520	64.46	31,605.90
F7	T4-W421	2024-07-07 T21:13	2024-07-08 T01:07	650.070	115.686	56,674.94
F7	T4-W421	2024-07-06 T15:34	2024-07-06 T17:47	650.720	69.41	33,970.21
F7	T4-W421	2024-07-05 T20:56	2024-07-05 T23:02	651.010	80.406	39,334.28
F7	T4-W421	2024-07-05 T05:24	2024-07-05 T10:07	651.300	126.474	61,843.00
F7	T4-W421	2024-07-03 T22:21	2024-07-04 T02:42	651.600	149.874	73,251.34

**Table 7:** The original data of roll T4-X303 on stand F5.

No	Roll	Time on Machine	Time off Machine	Diameter	Rolling Kilometerage	RUL
F5	T4-X303	2024-07-31 T23:30	2024-08-01 T02:07	684.270	48.984	22,798.02
F5	T4-X303	2024-07-30 T12:35	2024-07-30 T15:02	685.190	69.648	32,371.88
F5	T4-X303	2024-07-30 T02:30	2024-07-30 T05:42	685.610	102.704	47,706.83
F5	T4-X303	2024-07-29 T13:35	2024-07-29 T15:24	686.050	59.976	27,841.46
F5	T4-X303	2024-07-29 T02:48	2024-07-29 T04:52	686.620	56.05	25,997.38
F5	T4-X303	2024-07-28 T14:46	2024-07-28 T17:22	687.100	60.644	28,108.54
F5	T4-X303	2024-07-27 T21:50	2024-07-28 T00:32	687.540	82.03	37,996.63
F5	T4-X303	2024-07-27 T05:09	2024-07-27 T08:07	687.640	74.188	34,359.19
F5	T4-X303	2024-07-26 T17:08	2024-07-26 T19:07	688.500	51.336	23,745.89

**Table 8:** Comparison between the predicted RUL and the actual average RUL.

Roll/Stand	Predicted RUL	Actual Average RUL	Prediction Error (%)
T4-W311/F7	49,634.10	46,775.64	5.76
T4-W421/F7	48,245.54	45,786.72	5.10
T4-X303/F5	33,246.87	31,214.34	6.11

**Table 9:** Prediction error of models.

Model	RMSE	CD	MAE
The proposed method	1054.31	0.94	821.85
The exponential Wiener process	7253.82	0.42	5732.46
The RVM model based on Gaussian kernel	1793.43	0.78	1432.08

## Data Availability

The data cannot be made publicly available upon publication due to the presence of sensitive personal information and confidential corporate details. Nevertheless, the data supporting the findings of this study are available upon reasonable request from the authors.
